# Autonomic Control of the Heart and Its Clinical Impact. A Personal Perspective

**DOI:** 10.3389/fphys.2020.00582

**Published:** 2020-06-12

**Authors:** Maria Teresa La Rovere, Alberto Porta, Peter J. Schwartz

**Affiliations:** ^1^Department of Cardiology, IRCCS Istituti Clinici Scientifici Maugeri, Montescano (Pavia), Italy; ^2^Department of Biomedical Sciences for Health, University of Milan, Milan, Italy; ^3^Department of Cardiothoracic, Vascular Anesthesia and Intensive Care, IRCCS Policlinico San Donato, Milan, Italy; ^4^Center for Cardiac Arrhythmias of Genetic Origin and Laboratory of Cardiovascular Genetics, Istituto Auxologico Italiano, IRCCS, Milan, Italy

**Keywords:** heart rate variability, QT interval, baroreflex sensitivity, autonomic nervous system, sympathetic nervous system, vagal activity, long QT syndrome, sudden death

## Abstract

This essay covers several aspects of the autonomic control of the heart, all relevant to cardiovascular pathophysiology with a direct impact on clinical outcomes. Ischemic heart disease, heart failure, channelopathies, and life-threatening arrhythmias are in the picture. Beginning with an overview on some of the events that marked the oscillations in the medical interest for the autonomic nervous system, our text explores specific areas, including experimental and clinical work focused on understanding the different roles of tonic and reflex sympathetic and vagal activity. The role of the baroreceptors, not just for the direct control of circulation but also because of the clinical value of interpreting alterations (spontaneous or induced) in their function, is discussed. The importance of the autonomic nervous system for gaining insights on risk stratification and for providing specific antiarrhythmic protection is also considered. Examples are the interventions to decrease sympathetic activity and/or to increase vagal activity. The non-invasive analysis of the RR and QT intervals provides additional information. The three of us have collaborated in several studies and each of us contributes with very specific and independent areas of expertise. Here, we have focused on those areas to which we have directly contributed and hence speak with personal experience. This is not an attempt to provide a neutral and general overview on the autonomic nervous system; rather, it represents our effort to share and provide the readers with our own personal views matured after many years of research in this field.

## Introduction

One of the characteristics of the autonomic nervous system is the waxing and waning of its activity, both afferent and efferent. Similarly, the last 50 years have witnessed the waxing and waning of its interest for clinical cardiologists dealing with cardiac arrhythmias, sudden death, and heart failure.

Especially in the 1970s, but also later, much effort was devoted to the study of neural activity through the recording of single fibers in the sympathetic and vagal nerves (Malliani et al., 1969, 1973; Kunze, 1972; Cerati and Schwartz, 1991), which allowed the description of important autonomic reflexes (Schwartz et al., 1973). In the 1970s, 1980s, and 1990s, interest for the autonomic nervous system peaked. Some investigators focused on the stimulation of nerves directed to the heart trying to derive information for potential clinical translation (Schwartz, 1985), others focused on various aspects of the analysis of heart rate, either at rest (Kleiger et al., 1987) or in response to stimuli (Billman, 2009); these analyses of tonic or reflex autonomic activity had post-myocardial infarction (post-MI) and heart failure risk stratification as one significant objective (Schwartz et al., 1992a; Mortara et al., 1997; La Rovere et al., 1998, 2003). During the last 20 years, there was a surge of interest for the possibility of modulating autonomic activity, especially the vagal one, also in chronic conditions such as heart failure (Schwartz et al., 2008a, 2015; De Ferrari et al., 2011); however, the combination of unsatisfactory results and superficial analyses (Hauptman et al., 2012; Gold et al., 2016) has somewhat cooled down these hopes. One area where clinical success has fueled interest is the one related to the prevention of life-threatening events by cardiac sympathetic denervation (Schwartz, 2014).

Here, the three of us, who have collaborated in a number of studies, present our unabashed views on some of these topics.

## Initial Overview

The neural control of the heart is accomplished throughout a multilevel neural network within the central nervous system and peripheral extracardiac and cardiac ganglia that exert their influence *via* the sympathetic and parasympathetic nervous systems (Levy and Schwartz, 1994; Shivkumar et al., 2016). Cardiac diseases may profoundly affect central and peripheral mechanisms of neural control of cardiac function, thus resulting in maladaptive responses that may be critically involved in the progression of the disease or in the development of arrhythmias. Neural sensory information from the heart (Paintal, 1963; Schwartz et al., 1973), blood vessels, and other organs is processed at different levels within the neuraxis with a first level of integration represented by the intrinsic cardiac nervous system located in the cardiac ganglia within the heart. The intrinsic cardiac nervous system processes sensory information and provides efferent input to the myocardium under the tonic modulation of the extrinsic sympathetic and parasympathetic input. Arterial baroreceptors play a paramount role in the neural control of the cardiovascular system (Eckberg and Sleight, 1992). Arterial baroreceptors are stretch receptors embedded in the adventitia of the carotid sinus and aortic wall. Increases in arterial blood pressure will result in an increased rate of impulse firing to the nucleus tractus solitarius, which modulates sympathetic and parasympathetic output to the cardiovascular system. The baroreflex control of circulatory homeostasis occurs on a negative feedback basis. Thus, the attending reflex decrease in sympathetic activity and increase in vagal activity will reduce heart rate, cardiac contractility, and peripheral resistance. Opposite changes are associated with an arterial pressure decrease.

By complex interactions between the main neurotransmitters [namely, noradrenaline, acetylcholine, and neuropeptide Y; (Dusi et al., 2020)] and their effects on specific receptors of cardiac cells in the sinoatrial node, atrioventricular node, and left ventricle, the autonomic nervous system affects several aspects of cardiac electrophysiology. At the sinus node level, efferent vagal activity decreases while sympathetic activity increases the spontaneous depolarization rate of sinus node cells.

Furthermore, it has been known for many years that sympathetic nervous system stimulation may be pro-arrhythmic, particularly in conditions of acute myocardial ischemia (Harris et al., 1971; Schwartz and Vanoli, 1981; Janse et al., 1985; Schwartz et al., 1985), while vagal nerve stimulation may reduce the potential for lethal arrhythmias (Kent et al., 1973; Vanoli et al., 1991). Thus, by controlling the autonomic traffic to the heart, the baroreceptors are involved in the susceptibility to ventricular and supraventricular arrhythmias. Moreover, by controlling the hemodynamic adjustments to blood pressure changes, they also play a role in the clinical response to sustained rhythm disorders (De Ferrari et al., 1995; Landolina et al., 1997).

Damage to cardiac sensory nerve endings caused by acute myocardial infarction and left ventricular remodeling directly affects the baroreceptor system. The attending reflex autonomic dysfunction, characterized by reduced parasympathetic and increased sympathetic activity coupled with neural remodeling and nerve sprouting (Cao et al., 2000), promotes arrhythmogenesis.

The prominent neurohumoral mechanism at play in heart failure is the sympathetic nervous system whose increased activity coupled with vagal withdrawal is initiated by the arterial baroreflex (Hartupee and Mann, 2017). Although other mediators, including sympatho-excitatory reflexes, humoral factors, and central mechanisms (Floras and Ponikowski, 2015) contribute to the development of sympathetic-parasympathetic imbalance in heart failure, an impairment of baroreflex control of heart rate is a prominent characteristic of the heart failure syndrome and a reliable marker of the severity of the disease (Mortara et al., 1997).

Relevant insights into the pathophysiological implications of heart disease-related baroreflex impairment date back to the early 1970s when it was recognized that baroreceptor reflexes can be modulated by cardiac afferent sympathetic activity activated by mechanical and chemical stimuli (Malliani et al., 1973; Schwartz et al., 1973). An animal model provided the first evidence that reduction in cardiac parasympathetic control is associated with an increased risk for sudden death. In this canine model (Billman et al., 1982; Schwartz et al., 1984), baroreflex sensitivity (BRS) was impaired by myocardial infarction, with the greatest impairment noted in animals particularly susceptible to sudden death (Schwartz et al., 1988). Similarly in humans, a tight relationship between reduced baroreceptor activity and heart disease state was first reported by Eckberg et al. (1971) and was subsequently found to be associated with an increased risk of cardiac mortality and sudden cardiac death in post-MI and heart failure patients (Mortara et al., 1997; La Rovere et al., 1998, 2001).

The initial results with BRS led some investigators to consider the possibility that powerful baroreceptive reflex would imply that the attendant increase in vagal activity to the sinus node would extend to the ventricles as well. The high specificity of the cardiac innervation (Pagani et al., 1974; Randall, 1984), the central organization of cardiovascular reflexes (Wurster, 1984), and the important report by Inoue and Zipes (1987) indicate clearly that such an extrapolation would be both naïve and unwarranted. Indeed, a heart-rate response indicative of increased vagal activity does not exclude the possibility of a dominant sympathetic activity at ventricular level (e.g., the diving reflex, hypoxia, and inferior myocardial ischemia). Nonetheless, the most frequent reflex response is synergistic, i.e., one limb of the autonomic nervous system is excited with simultaneous inhibition of the other (Wurster, 1984), and the reduction in heart rate produced by the baroreflex is accompanied by a reflex withdrawal of sympathetic activity that is generalized and extends to the ventricles. In the sudden death animal model that played such an important role in the development of the clinical interest for BRS (Schwartz et al., 1984), the dogs with higher BRS were also those with larger heart rate reductions during acute myocardial ischemia despite continuation of exercise (Schwartz et al., 1984). One logical implication is that the animals responding with strong vagal reflexes to blood pressure increases are likely to respond similarly to acute myocardial ischemia. The animals with the greatest sinus node response to the baroreflex test are less prone to sudden death during myocardial ischemia, and conversely, those with the most reduced BRS are more vulnerable to ventricular fibrillation. This does not mean that the baroreflex test predicts the autonomic changes at the ventricular level during myocardial ischemia but indicates that it can often predict the outcome during an ischemic episode, which is what really matters. Although, as correctly stated (Inoue and Zipes, 1987), the use of spontaneous or reflex changes in heart rate as an indicator of what might happen at the ventricular level would certainly be naive, their use to identify individuals at varying risk of life-threatening events is a rational exploitation of the current understanding of cardiovascular pathophysiology.

## Assessment of Cardiac Autonomic Function

As the baroreflex affects the balance between parasympathetic inhibition and sympathetic excitation of the sinoatrial node of the heart, sinus node activity (either spontaneous or in response to a provocation) can provide information on the underlying regulatory system.

### Assessment of Arterial Baroreflex Control

Several methods have been developed so far to evaluate arterial baroreflex control in humans (La Rovere et al., 2008; Pinna et al., 2017). The reference method in clinical and research applications entails the assessment of the heart rate response to a physiological provocation (Smyth et al., 1969; La Rovere et al., 2008). In the original method, intravenous injections of small boluses of phenylephrine are used to raise blood pressure transiently, and the resultant reflex bradycardia (expressed as the following heart periods) is used as an index of BRS. A wealth of non-invasive indicators of the arterial-cardiac baroreceptor reflex sensitivity can be obtained by the joint analysis of beat-to-beat spontaneous fluctuations of systolic blood pressure and RR interval series (La Rovere et al., 2008). These methods include: model-free techniques (Robbe et al., 1987; Pinna et al., 2002), interactions among heart period and systolic arterial pressure (Porta et al., 2000; Nollo et al., 2005; Milan-Mattos et al., 2018), models searching for specific patterns of baroreflex origin (Bertinieri et al., 1985) or heart rate responses to systolic pressure changes (Bauer et al., 2010), and others merely requiring a certain degree of association between spontaneous heart period and systolic arterial pressure variations (Westerhof et al., 2004). Some methods lead to an indirect estimate of BRS *via* analysis of the bi-phasic response of the sinus node to a premature ventricular contraction (named heart rate turbulence) that is largely dependent on the baroreflex (Schmidt et al., 1999; La Rovere et al., 2011). Despite indices of baroreflex control derived from spontaneous variability cannot be considered fully equivalent to the interventional ones (Diaz and Taylor, 2006), their value in clinical setting has been proved (La Rovere et al., 2008; Pinna et al., 2017). The reliability of these non-invasive indices has been recently reviewed (Pinna et al., 2015) with special attention to their predictive value (Pinna et al., 2017).

### Assessment of Heart Rate Variability

Since the seminal study by Akselrod et al. (1981), autonomic function has been non-invasively inferred from the variability of sinus RR interval obtained from surface electrocardiogram (ECG). The disappearance of RR variability after vagal blockade by high dose atropine not only proved the predominance of vagal over sympathetic cardiac modulation in humans at rest (Pomeranz et al., 1985; Montano et al., 1998) but also confirmed that RR interval variability was related to autonomic control. Indeed, in humans at rest, the primacy of the vagal versus the sympathetic drive leads to a heart rate lower than the intrinsic heart rate of the isolated heart (Jose and Collison, 1970).

The RR mean provides an indication of the tonic balance between sympathetic and vagal mean neural activities (Malik et al., 2019b), while the magnitude of the RR variations about its mean is linked to the balance of the spontaneous variations of vagal and sympathetic neural activities about their correspondent means, usually referred to as vagal and sympathetic modulations (Task Force of the European Society of Cardiology and the North American Society of Pacing and Electrophysiology, 1996; Pagani et al., 1997; Bauer et al., 2017; Malik et al., 2019a). A number of techniques have been developed to quantify the RR interval variability in order to evaluate cardiac autonomic regulation. The measurement of RR interval variability was initially based on simple statistics, such as the standard deviation of RR interval variation and its derivative, and on power spectral analysis that separates and quantifies the various oscillations that exist in the RR interval signal. At variance with the conventional measures of RR interval variability, complexity markers and fractal measures of HRV account for the inherent irregularity, long range correlation, and scale invariance of the spontaneous fluctuations of RR interval (Goldberger, 1996; Porta et al., 2009).

It has been recently stressed that RR variability markers might be biased proxies of autonomic modulation as a result of the nonlinear relation between mean RR and magnitude of RR changes (Opthof et al., 1984; Boyett et al., 2019; Malik et al., 2019a). The rate-dependency of RR variability markers is the consequence of the direct effect of acetylcholine concentration on the diastolic depolarization rate of the sinus node pacemaker cells producing larger variations of the cycle length if the cycle length is longer (Zaza and Lombardi, 2001). This relation might limit the value of RR variability markers expressed in absolute units because an augmented modulation of the autonomic activity increases RR variability indices but a greater fluctuations of RR might be simply a consequence of bradycardia, regardless of whether it is of autonomic origin or due to modifications of the properties of sinus node pacemaker cells [e.g., I_f_ modifications; Zaza and Lombardi, (2001); Da Silva et al. (2015); Boyett et al. (2019)]. Since this effect is the mere consequence of the sinus node transduction process, it could affect any marker based on RR changes including BRS. Therefore, the possibility of interpreting RR variability markers as proxies of autonomic modulation is fully preserved as long as the compared populations and/or experimental conditions exhibit the same RR mean. Alternatively, it was suggested to use RR variability indices that feature an intrinsic normalization (Zaza and Lombardi, 2001; Da Silva et al., 2015). Among those indices, normalized high frequency (HF) powers and the low frequency (LF)/HF ratio can be exploited (Pagani et al., 1997). Especially whether the RR mean varies among groups and experimental conditions and, thus, the genuine role of an altered neural modulation is not warranted, it is recommended to check for the potential variations of the LF/HF ratio before concluding that RR variability indices expressed in absolute units indicate modifications of the autonomic control.

### Concurrent Assessment of RR and QT Variability

The difficulty in the assessment of the sympathetic modulation from RR variability is a direct consequence of the vagal nature of the spontaneous fluctuations of RR (Eckberg, 1997), especially when the magnitude of the RR changes is assessed in absolute units (Montano et al., 1994). This observation, in association with the clinical importance of the non-invasive inference of cardiac sympathetic modulation, has led to search for possible alternatives, still obtained from the ECG, to the sole analysis of RR variability.

An important one, in our opinion, is the study of the QT interval variability (Malik, 2008; Berger, 2009; Baumert et al., 2016). Its interest lies in the fact that the amplitude of the QT changes has been related to the magnitude of sympathetic control. Indeed, the higher the sympathetic drive and its variations about its mean value such as during an orthostatic challenge, mental stress, or advanced age, the greater the magnitude of the QT variations in healthy individuals (Negoescu et al., 1997; Porta et al., 1998a, 2010, 2011; Yeragani et al., 2000a; Piccirillo et al., 2001, 2006; Boettger et al., 2010; Baumert et al., 2016; El-Hamad et al., 2019). This link holds even in pathological conditions characterized by a high sympathetic drive (Berger et al., 1997; Yeragani et al., 2000b; Bär et al., 2007; Baumert et al., 2008, 2011) and provides new clues for stratifying the risk of arrhythmic events (Atiga et al., 1998; Piccirillo et al., 2007; Segerson et al., 2008; Chen et al., 2011; Dobson et al., 2011; Oosterhoff et al., 2011; Tereshchenko et al., 2012; Porta et al., 2015).

These observations suggested a possible strategy to separately quantify vagal and sympathetic modulations in humans *via* the concomitant analysis of RR and QT variabilities (Porta et al., 2015). Vagal modulation is inferred from the respiratory sinus arrhythmia, namely the portion of the RR variability in the HF (from 0.15 to 0.5 Hz) band (Hirsch and Bishop, 1981; Pomeranz et al., 1985). Sympathetic modulation is inferred from the power of the QT variability in the LF (from 0.04 to 0.15 Hz) band (Porta et al., 2011; Baumert et al., 2016). This choice is more robust than the mere exploitation of the QT variance because it prevents the bias produced by non-autonomic influences such as cardiac axis movements leading to periodical artifacts which would affect the QT measurement at the respiratory rate (Lombardi et al., 1996; Porta et al., 1998b). The interpretation of QT variability markers is made more complex by the QT-RR relation (Bazett, 1920), which mirrors on the surface ECG the adaptation of action potential duration to the cycle length observed at the cellular level (Conrath and Opthof, 2006), and by the influences of the autonomic nervous system on the QT-RR relation (Zaza et al., 1991; Porta et al., 1998a; Magnano et al., 2002). Modeling approaches can describe the dynamic dependence of QT on previous RR variations (Zaza et al., 1991; Porta et al., 1998a, 2010) and even account for confounding factors such as respiration (Porta et al., 2017). Alternative approaches excluding the influences of cardiac neural control directed to the sinus node on the regulation of the QT dynamics and preventing the need of hypothesizing any *a priori* defined, and arbitrary, QT-RR relation (Pueyo et al., 2004) are based on gating the QT variability analysis at similar RR mean (Browne et al., 1983) or on the normalization of QT variability markers to the magnitude of RR changes (Berger et al., 1997; Baumert et al., 2016).

### Complexity of the Cardiac Autonomic Control

Complexity analysis is an additional approach for the assessment of cardiac control with an inherent normalization given that it is fully independent of the amplitude of spontaneous RR and QT changes (Pincus and Goldberger, 1994; Porta et al., 2009). Under normal conditions, the simultaneous action of multiple regulatory mechanisms operating with slightly different frequencies within the LF and HF bands produces irregular changes of RR and QT intervals. Disease and aging impair the sinus node responsiveness and decrease the level of irregularity of the RR and QT beat-to-beat dynamics (Goldberger, 1996). Complexity analyses of RR and QT variabilities provide non-redundant information. Indeed, the larger irregularity of the QT variability compared to that of the RR variability points to the greater complexity of the neural control directed to the ventricles than that to the sinus node (Inoue and Zipes, 1987; Lewis and Short, 2007; Baumert et al., 2012; Bari et al., 2014a). The decreased complexity of the RR variability during vagal withdrawal and sympathetic activation induced by orthostatic challenge (Porta et al., 2007; Turianikova et al., 2011; Baumert et al., 2014) is interpreted as a consequence of the reduction of the respiratory sinus arrhythmia and of the increase of a dominant LF component limiting the spectral content of the RR variability series (Porta et al., 2012). Therefore, complexity indices derived from RR variability are mainly under vagal control (Porta et al., 2012). Indeed, low-pass filtering approach canceling respiratory sinus arrhythmia from the RR variability prevented the increase of RR variability complexity during nighttime and under β-blockers (Bari et al., 2014a). At difference with the complexity of RR variability, the complexity of the QT variability in healthy individuals during orthostatic challenge and in pathological populations featuring a dominant sympathetic drive remains high (Baumert et al., 2014; Li et al., 2019) or even increases (Sosnowski et al., 2001; Nahshoni et al., 2004; Porta et al., 2010; Li et al., 2015) compared to basal condition or control subjects. Senescence in a healthy population is accompanied by an increase of QT variability complexity (Boettger et al., 2010). The dynamics of QT variability become more irregular during sympathetic activation due to the prevailing action of inputs driving QT independently of RR changes (Porta et al., 2010). The decrease of the T-wave amplitude with sympathetic activation is likely to play a role in increasing the beat-to-beat irregularity of QT by making the process of delineation of the T-wave offset more difficult (Baumert et al., 2016). Therefore, the complexity of the QT variability could largely represent the sympathetic control directed to the ventricles, largely unrelated to the cardiac autonomic regulation impinging on the sinus node. We suggested that a limited complexity of the QT variability might be protective against arrhythmic risk (Bari et al., 2014a,b).

## Risk Stratification

Effective risk stratification for patients who might develop life-threatening ventricular arrhythmia and sudden cardiac death is one of the main unsolved areas in clinical cardiology. Arrhythmic risk represents the sum of several different risk-augmenting processes and factors. Understanding the relation between changes in autonomic activity and cardiac electrophysiological properties has led to the view that the autonomic nervous system modulates interactions between triggering factors and the underlying electrophysiologic substrate. This points to a significant potential prognostic value of markers of autonomic activity.

Since the 1990s, the analysis of BRS has been considered as a tool that might help identifying “high-risk” patients. A multicenter study on more than 1,200 post-infarction patients demonstrated the incremental prognostic value provided by an impaired BRS when combined to left ventricular ejection function and to the potential trigger of non-sustained ventricular tachycardia (La Rovere et al., 1998, 2001). Specifically, a depressed BRS, a reduced left ventricular ejection fraction, and the presence of non-sustained ventricular tachycardia were all independent predictors of mortality, but depressed BRS almost doubled the risk of death provided by the other two markers. Moreover, among patients with either reduced or preserved left ventricular function but without signs of electrical instability, mortality differed significantly according to the presence or absence of preserved autonomic function (La Rovere et al., 2001; De Ferrari et al., 2007).

The role of baroreflex-mediated responses in the control of hemodynamic stability is particularly relevant during the course of a sustained ventricular rhythm. Inadequate baroreflex-mediated sympatho-excitation during a sustained ventricular tachycardia in post-infarction patients was the leading cause of an unfavorable hemodynamic profile leading to circulatory collapse (Landolina et al., 1997).

Randomized trials, demonstrating that among post-infarction patients mortality can be effectively reduced by prophylactic implantation of a cardioverter defibrillator, established a paradigm shift in risk stratification through the assessment of left ventricular ejection fraction as the gold standard risk predictor. However, this does not deprive autonomic markers of their clinical value (Wellens et al., 2014). It is now clear that left ventricular ejection fraction measurement has both limited sensitivity and specificity as a tool for arrhythmic risk stratification and that the field of risk stratification should move from the “high-risk ejection fraction” to the broader concept of the “high-risk patients” (Chugh, 2017). This transition implies a novel opportunity for autonomic markers to be re-evaluated in their involvement in the pathogenesis of arrhythmic risk and incorporated in novel prediction models. Moreover, novel ECG-based risk markers that quantify sympathetic activity-associated repolarization instabilities are promising in their ability to guide decisions about the prophylactic implantation of a cardioverter defibrillator (Bauer et al., 2019). The markers tested by Bauer et al. (2019) are framed in an emerging area of biomedical signal processing aiming at monitoring relevant electrocardiographic fiducial points and time intervals under the hypothesis such that their evolution over time might provide information about cardiac control.

In a founder population of long QT syndrome type 1 (LQT1), which avoids the confounding factors due to different mutations and segregates the malignant *KCNQ1*-A341V mutation (Brink et al., 2005; Crotti et al., 2007; Brink and Schwartz, 2009), the characterization of cardiac autonomic control and baroreflex function was found to be useful to improve the risk stratification of arrhythmic events (Schwartz et al., 2008b; Crotti et al., 2012; Bari et al., 2014a,b, 2015; Porta et al., 2015). In this population which is at the highest risk of fatal events in situations of high sympathetic drive (Schwartz et al., 2001), it was found that subjects who did not experience arrhythmic events, namely the asymptomatic mutation-carriers, have a completely different autonomic profile compared to those experiencing syncope or cardiac arrest requiring resuscitation. Indeed, asymptomatic individuals exhibited longer RR (Schwartz et al., 2008b), lower BRS (Schwartz et al., 2008b), higher QT variability in the LF band during daytime (Porta et al., 2015), lower respiratory sinus arrhythmia during nighttime and under β-blockers (Porta et al., 2015), slower heart rate recovery after exercise test (Crotti et al., 2012), and lower QT variability complexity (Bari et al., 2014a,b, 2015). These findings suggested that, besides RR lengthening, the combination of a more reactive sympathetic drive to the ventricles (i.e., adapting more rapidly QT to RR changes and limiting irregularity of QT changes during a sympathetic stressor) and of a sluggish vagal responsiveness after exercise represents a protective mechanism. Remarkably, non-mutation carriers belonging to the same family line (Brink et al., 2005; Brink and Schwartz, 2009) have an autonomic profile more similar to symptomatic patients than asymptomatic ones, thus suggesting that there are peculiar traits of the autonomic control that might be key for survival because they reduce the severity of the disease (Schwartz et al., 2008b; Porta et al., 2015).

## Neuromodulation

### Vagal Neuromodulation

One relevant aspect of several abnormalities related to the baroreceptors and autonomic nervous system pathophysiology is that they are often correctable by treatment. While β-blockers are the mainstay in the management of autonomic imbalance, device technology and advances in neuromodulatory techniques paved the way to directly target the autonomic nervous system. Baroreflex activation therapy (BAT), providing chronic baroreflex activation through electrical stimulation of the carotid sinus, has been initially developed for the treatment of resistant hypertension. Clinical studies have underlined the potential of BAT to improve blood pressure control and reduce the need of anti-hypertensive therapy at cost of few side effects despite the invasiveness of the procedure (Bolignano and Coppolino, 2018). BAT is currently being evaluated in heart failure with reduced ejection fraction. Initial studies support the hypothesis that baroreflex activation can add significant therapeutic benefit on top of guideline-directed medical therapy in patients with advanced heart failure. A randomized controlled trial (the BeAT-HF trial) is actively recruiting an estimated sample size of 480 patients with New York Heart Association functional class II heart failure but excluding patients actively receiving cardiac resynchronization therapy; its completion is expected by April 2021 (Mann and Abraham, 2019).

Experimental studies in an established conscious canine model of post-MI sudden cardiac death (Schwartz et al., 1984) demonstrated that vagus nerve stimulation (VNS) was effective in preventing ventricular fibrillation induced by acute myocardial ischemia (Vanoli et al., 1991). The initially promising translation of animal studies to the clinical setting of patients with HF and reduced ejection fraction (Schwartz et al., 2008a; De Ferrari et al., 2011) did not show consistent results in randomized trials (Zannad et al., 2015; Gold et al., 2016). In the debate following these studies, several critical issues (patient selection, proper titration of VNS therapy, effective markers for therapy, and pattern of vagal fibers stimulation) have been identified that would require further assessment.

Transcutaneous electrical stimulation of the auricular branch of the vagus nerve located at the tragus, which is effective in stimulating afferent vagal nerve fibers, has been suggested to represent an alternative access path to the same neuronal network without invasiveness and common side effects including hoarseness, sore throat, shortness of breath, and coughing, even though it is likely to lead to a smaller release of ACh compared to direct vagal efferent stimulation. In a study based on spontaneous variability, in young healthy subjects, transcutaneous VNS acutely reduced resting heart rate and the response to orthostatic stress (Tobaldini et al., 2019). Transcutaneous VNS is being studied for a number of pathological conditions including ventricular arrhythmias, heart failure, and myocardial infarction. Experimental and clinical data recently suggested that chronic intermittent VNS lasting 2 h/day for 2 months reduced inducibility of ventricular arrhythmias (Zhu et al., 2019).

### Sympathetic Neuromodulation

The fact that acute myocardial ischemia is often associated with life-threatening arrhythmias was recognized from the early days (Harris et al., 1971). When it was shown that acute myocardial ischemia also elicits a powerful excitatory sympathetic reflex within seconds (Malliani et al., 1969), thus increasing the release of norepinephrine at the ventricular level, the link was established. One obvious consequence was the rationale for the use of β-adrenergic blocking agents to prevent cardiac arrhythmias in ischemic heart disease. Another consequence was the concept that if arrhythmias are triggered by an abrupt release of norepinephrine, the section of the nerves mediating this release might have had a protective effect (Schwartz, 2014).

Given the quantitative dominance of the left sided cardiac sympathetic nerves at the ventricular level, the interest went immediately to the potential effects of left cardiac sympathetic denervation (LCSD). Thus, a series of experiments, mostly performed in the 1970s, provided the necessary information. It was shown that LCSD prevents arrhythmias associated with acute myocardial ischemia in normal hearts (Schwartz et al., 1976b) and in hearts with a healed myocardial infarction (Schwartz and Stone, 1980), that it does not impair cardiovascular performance during exercise (Schwartz and Stone, 1979), that it does increase the capability of the coronary bed to dilate (Schwartz and Stone, 1977), and that it does not cause denervation supersensitivity (Schwartz and Stone, 1982). However, the most important effect in terms of clinical relevance is the increase produced by LCSD on the threshold for ventricular fibrillation (Schwartz et al., 1976a). This makes it less likely that a heart will fibrillate and, together with the overall reduction in the norepinephrine release, constitutes the primary rationale for the use of LCSD in several conditions in which the risk for ventricular fibrillation is high.

The evidence for a powerful antifibrillatory effect of LCSD is now firmly established (Schwartz, 2014) and has been observed at clinical level in three different sets of patients: post-MI patients at high risk for sudden death (Schwartz et al., 1992b), long QT syndrome patients (Schwartz et al., 2004), and patients with catecholamine polymorphic ventricular tachycardia (CPVT) syndrome (De Ferrari et al., 2015). Whenever there is a recurrence after LCSD, which is not common for LQTS and CPVT patients, it is reasonable to perform right cardiac sympathetic denervation as well, as we started to do in the 1990s (Schwartz et al., 1991, 2004). There are growing data suggesting that bilateral cardiac sympathetic denervation can be useful in patients with recurrent ventricular tachycardia related to either ischemic heart disease or dilated cardiomyopathy (Vaseghi et al., 2014). Different views exist on the timing for ablating the right cardiac sympathetic nerves; namely, whether together with the left or just in case of failure of unilateral left cardiac sympathetic denervation. Our view is to follow the time-honored precepts of medicine which suggest to begin by the lowest effective dose and to increase it whenever this fails. An implication is that for a number of patients, unilateral left cardiac sympathetic denervation will be sufficient (Schwartz, 2014).

Overall, it is now clear that cardiac sympathetic denervation can save lives while preserving adequate quality of life (Antiel et al., 2016; Schwartz, 2016) and that there is not always the need to rush toward an implantable cardioverter defibrillator.

## Renal Denervation

Renal sympathetic nerve activity plays a crucial role in the control of cardiovascular homeostasis and is involved not only in the pathogenesis of hypertension but also in other cardiovascular processes such as heart failure, and perhaps cardiac arrhythmias. Renal afferent and efferent nerves function in a reflex loop where afferent input from the kidney to the central nervous system is integrated with inputs from other neural reflexes to determine the level of sympathetic outflow to individual organs. Renal denervation (RDN) as a method of modulating sympathetic activity by interrupting afferent and efferent sympathetic nerve signaling appears to be an attractive therapeutic target in patients with cardiovascular disease triggered by sympathetic overactivity such as hypertension, heart failure, and – according to some – even atrial or ventricular arrhythmias. RDN has been initially introduced to reduce blood pressure in subjects with resistant hypertension (Mahfoud et al., 2013). While initial clinical trial results failed to reach a consensus on the efficacy of RDN in this context (Bhatt et al., 2014), three subsequent sham-controlled studies that were carefully designed and rigorously conducted have shown that RDN significantly reduces blood pressure regardless of the use of antihypertensive drugs (Townsend et al., 2017; Azizi et al., 2018; Kandzari et al., 2018). Notably, the use of multi-electrode catheter and more ablations per artery have definitely improved the RDN procedure in the more recent studies that also took into account the distribution of the sympathetic nerves among the renal arteries.

Clinical implications of RDN are well beyond blood pressure control. Interestingly, a recent meta-analysis including 17 studies revealed that RDN improved a number of cardiovascular markers of organ damage including left ventricular mass index, central augmentation index, and carotid-femoral pulse wave velocity independent of blood pressure (Kordalis et al., 2018). Moreover, several clinical RDN studies report beneficial effects on ventricular and supraventricular arrhythmias (Ukena et al., 2012; Pokushalov et al., 2014). In the recently reported ERADICATE-AF trial that randomized 302 patients with paroxysmal atrial fibrillation and hypertension, RDN added to catheter ablation, compared with catheter ablation alone, significantly increased the likelihood of freedom from atrial fibrillation at 12 months (Steinberg et al., 2020). While we report these studies and their claims, we cannot help expressing a certain degree of skepticism on the rationale by which RD should provide protection against life-threatening cardiac arrhythmias. The underlying concept seems to be that reduction or elimination of renal afferent would decrease the efferent sympathetic activity directed to the heart, which is mediated by the stellate ganglia. We find it difficult to conceive how this “reduction” could be greater than that produced by the direct section of these nerves, as it is produced by the cardiac sympathetic denervation performed by removing the lower half of the stellate ganglion/ganglia with the first four thoracic ganglia.

RDN has been reported to exert beneficial effects on cardiac function and remodeling in animal models of heart failure (Sharp et al., 2018), but the results in patients are largely inconsistent due in part to limited power with small sample sizes. In a meta-analysis including two controlled (80 patients) and two uncontrolled studies (21 patients) (Fukuta et al., 2017), 6 months after RDN, there was a greater increase in left ventricular ejection fraction and a greater decrease in left ventricular end-diastolic diameter in the RDN group than in the control group. No serious adverse events such as acute renal artery stenosis and dissection occurred.

## Conclusions

A paper like this one does not really need a traditional conclusion, which would merely be a pale summary of what has been a serious effort to share with the interested reader our experience and our views. Our hope is that more and more young investigators will be attracted by this fascinating field of research, which is endowed with so many areas of clinical relevance.

## Author Contributions

PS, AP, and MR contributed to the conception and design of research, drafted the manuscript, edited and revised critically the manuscript, and approved the final version of the manuscript.

### Conflict of Interest

The authors declare that the research was conducted in the absence of any commercial or financial relationships that could be construed as a potential conflict of interest.
